# LECT2: A pleiotropic and promising hepatokine, from bench to bedside

**DOI:** 10.1111/jcmm.17407

**Published:** 2022-06-03

**Authors:** Yuan Xie, Kai‐Wei Fan, Shi‐Xing Guan, Yang Hu, Yi Gao, Wei‐Jie Zhou

**Affiliations:** ^1^ General Surgery Center, Department of Hepatobiliary Surgery II, Guangdong Provincial Research Center for Artificial Organ and Tissue Engineering, Guangzhou Clinical Research and Transformation Center for Artificial Liver, Institute of Regenerative Medicine Zhujiang Hospital, Southern Medical University Guangzhou China; ^2^ Department of General Surgery II The First People's Hospital of Zhaoqing Zhaoqing China; ^3^ Department of Cerebrovascular Disease The First People's Hospital of Zhaoqing Zhaoqing China; ^4^ Department of Pathology, School of Basic Medical Sciences Southern Medical University Guangzhou China; ^5^ Department of General Surgery & Guangdong Provincial Key Laboratory of Precision Medicine for Gastrointestinal Tumor, Nanfang Hospital, First Clinical Medical College Southern Medical University Guangzhou China

**Keywords:** biomarker, hepatokine, LECT2, target

## Abstract

LECT2 (leucocyte cell‐derived chemotaxin 2) is a 16‐kDa protein mainly produced by hepatocytes. It was first isolated in PHA‐activated human T‐cell leukaemia SKW‐3 cells and originally identified as a novel neutrophil chemotactic factor. However, many lines of studies suggested that LECT2 was a pleiotropic protein, it not only functioned as a cytokine to exhibit chemotactic property, but also played multifunctional roles in some physiological conditions and pathological abnormalities, involving liver regeneration, neuronal development, HSC(haematopoietic stem cells) homeostasis, liver injury, liver fibrosis, hepatocellular carcinoma, metabolic disorders, inflammatory arthritides, systemic sepsis and systemic amyloidosis. Among the above studies, it was discovered that LECT2 could be a promising molecular biomarker and therapeutic target. This review summarizes LECT2‐related receptors and pathways, basic and clinical researches, primarily in mice and human, for a better comprehension and management of these diseases in the future.

## 1 INTRODUCTION

LECT2 (leucocyte cell‐derived chemotaxin 2) was first isolated by Yamagoe et al in PHA‐activated human T‐cell leukaemia SKW‐3 cells and originally identified as a novel neutrophil chemotactic factor, in 1996.^1^ The human LECT2 gene has been located to chromosome 5q31.1–32, comprising three introns and four exons encoding a 151 amino acid protein, with a molecular mass of approximately 16‐kDa.^2^ LECT2 protein belongs to the zinc‐dependent metalloendopeptidases M23 family (PF01551), featured by a possession of a zinc ion as a cofactor and a preference for peptides containing polyglycine residues, noteworthily, it is the only vertebrate protein in the M23 family.^3^, ^4^, ^5^ Although LECT2 is mainly synthesized and secreted to bloodstream by hepatocytes,^6^ it is also found in presence of other cells, such as vascular endothelial cells, smooth muscle cells, cerebral nerve cells and adipocytes.^7^ Accumulating evidence demonstrated that the mammalian LECT2 was a pleiotropic protein, it not only acted as a cytokine to exhibit chemotactic property, but also played multifunctional roles in several physiological conditions and pathological abnormalities, involving liver regeneration, neuronal development, HSC (haematopoietic stem cells) homeostasis, liver injury, liver fibrosis, hepatocellular carcinoma, metabolic disorders, inflammatory arthritides, systemic sepsis and systemic amyloidosis. In this review, we summarize LECT2‐related receptors and pathways (Table 1), basic and clinical researches (Table 2), primarily in mice and human, for a better comprehension and management of these diseases in the future.

**TABLE 1:** Interaction, pathway and effect between LECT2 and its receptors

Ligand	Receptor	Pathway	Natural interaction and effect	References
LECT2	Tie1	MAPK/PPAR‐γ↑	To inhibit portal angiogenesis, promote liver sinusoid capillarization and worsen liver fibrosis	16
	VEGFR2	ERK/AKT↓	To suppress endothelial cell proliferation, migration, tube formation and HCC growth	24
	MET	RAF‐1/ERK↓	To block the vascular invasion and tumour metastasis of HCC	25
	CD209	JNK↑	To promote the expressions of intercellular adhesion molecule‐1 and pro‐inflammatory cytokines in HUVECs and THP‐1 cells	33
	CD209	P38/NF‐κB↑	To induce inflammatory response and insulin resistance in 3T3‐L1 adipocytes	34
	CD209a	Raf‐1/NF‐κB↑	To enhance bacterial killing and nitric oxide production in the RAW264.7 macrophages	39
	CD209a	C3/CR3↑	To increase phagocytic and bacterial killing activities of the macrophages	40

**TABLE 2:** Major basic and clinical researches concerning LECT2

Author	Year	Country	Study type	Subject	Main point	References
Yamagoe S	1996	Japan	Basic research	Cells	LECT2 was a 16‐KD protein with chemotactic activity for human neutrophils.	1
Segawa Y	2001	Japan	Basic research	Mice	LECT2 was found inversely related to the expressions of TNF‐α and IFN‐γ in the injured liver.	8
Ohtomi M	2007	Japan	Basic research	Mice	LECT2 may trigger the early events of hepatic regeneration.	9
Sato Y	2004	Japan	Clinical research	Patients	Serum LECT2 level may be a prognostic indicator for acute liver failure.	10
Koshimizu Y	2010	Japan	Basic research	Mice	LECT2 regulates the extension of axons and dendrites and the expressions of NGF, BDNF and NT‐3 during neuronal development	11
Koshimizu Y	2010	Japan	Basic research	Mice	LECT2 regulates neuritic extension by microtubular morphallaxis through katanin‐P60.	12
Lu XJ	2016	China	Basic research	Mice	LECT2 is an extra‐medullar cytokine that contributes to HSC homeostasis.	13
Saito T	2004	Japan	Basic research	Mice	LECT2 might regulate the homeostasis of NKT cells in the liver and might be involved in the pathogenesis of hepatitis.	14
Okumura A	2017	Japan	Basic research	Mice	LPS/D‐GalN‐induced liver injury could be alleviated in LECT2 deficient mice, due to reduced IFN‐γ production from NK and NKT cells.	15
Xu M	2019	China	Basic and clinical research	Mice and patients	LECT2 may be a potential biomarker and therapeutic target for liver fibrosis.	16
Lin Y	2021	China	Basic research	Mice	The combination of AAV9‐LECT2 small hairpin RNA and bevacizumab could significantly improve the therapeutic effects on liver fibrosis.	17
Xu H	2021	China	Clinical research	Patients	LECT2 could act as a direct biomarker to predict liver fibrosis more accurately.	18
Sak JJ	2021	Poland	Clinical research	Patients	LECT2 might be used as a diagnostic and monitoring biomarker for alcohol‐induced liver cirrhosis.	19
Ovejero C	2004	France	Basic and clinical research	Mice and patients	LECT2 is a direct target gene of Wnt/β‐catenin signalling in the liver.	20
Uchida T	1999	Japan	Clinical research	Patients	Weaker LECT2 expression went along with the progression of multistep hepatocarcinogenesis.	21
L'Hermitte A	2018	France	Basic and clinical research	Mice and patients	LECT2 plays a key role in the liver tumorigenesis and it may be a promising immunotherapeutic option for HCC.	22
Okabe H	2014	USA	Basic and clinical research	Mice and patients	Serum LECT2 could be a potential biomarker for HCC.	23
Chen CK	2016	China	Basic research	Cells and Mice	LECT2 selectively suppressed VEGF_165−_ induced HCC angiogenesis.	24
Chen CK	2014	China	Basic and clinical research	Mice and patients	LECT2 inhibited HCC growth by the direct binding and inactivation of MET receptor.	25
Ong HT	2011	Singapore	Basic and clinical research	Mice and patients	Reduced LECT2 expression closely correlated with early recurrence and poor prognosis in HCC patients.	26
Chikamoto K	2016	Japan	Basic research	Mice	Serum LECT2 levels showed a positive correlation with liver triglyceride contents.	27
Tanisawa K	2017	Japan	Clinical research	Patients	Plasma LECT2 levels exhibited a strong relationship with visceral fat area in human.	28
Okumura A	2013	Japan	Clinical research	Patients	LECT2 is a novel obesity‐related protein and serum LECT2 levels are increased by obesity and fatty liver.	29
Lan F	2014	Japan	Basic research	Cells and Mice	LECT2 may be a therapeutic target for obesity‐associated insulin resistance.	30
Hwang HJ	2015	Korea	Basic research	Cells and Mice	Gemigliptin might alleviate hepatic steatosis and insulin resistance by supressive LECT2 expression.	31
Takata N	2021	Japan	Basic research	Cells and Mice	LECT2 expressions positively correlated with hepatic infammation and steatosis, it contributed to M1‐like macrophage phenotype and the M1/M2 ratio.	32
Hwang HJ	2015	Korea	Basic research	Cells	LECT2 might directly mediate in the atherosclerotic inflammatory reactions in human endothelial cells.	33
Jung TW	2018	Korea	Basic research	Cells	LECT2 stimulates inflammatory response and insulin resistance in adipocytes via CD209/P38 dependent pathway.	34
Kameoka Y	2000	Japan	Clinical research	Patients	There was a clear link between LECT2 genotype and incidence and severity of RA.	35
Okumura A	2008	Japan	Basic research	Mice	LECT2 directly suppresses the development of CAIA.	38
Shen HX	2016	China	Basic research	Cells and Mice	LECT2 can enhance Helicobacter pylori killing and nitric oxide production in macrophages.	39
Lu XJ	2013	China	Basic research	Cells and Mice	LECT2 enhanced the function of macrophages via CD209a receptor.	40
Dang MH	2010	Japan	Basic research	Mice	LECT2 enabled the wild‐type septic mice to survive longer via down‐regulation of TNF‐α and IL‐6.	41
Ando K	2012	Japan	Clinical research	Patients	LECT2 concentrations correlated with the severity of systemic inflammation in patients with sepsis.	42
Mereuta OM	2014	USA	Clinical research	Patients	LECT2 amyloidosis is a common cause of hepatic amyloidosis in the USA.	43
Benson MD	2008	USA	Clinical research	Patients	LECT2 was identified as a new amyloidogenic protein in renal amyloid deposits.	45
Ha JH	2021	USA	Basic research	protein	I40V mutation together with zinc deficiency destabilize the structure of LECT2 and increase the aggregation tendency.	52

### 1.1 Liver regeneration

LECT2 may be involved in the early period of liver regeneration. In concanavalin A (ConA)‐induced hepatic injury model, both LECT2 mRNA and protein levels decreased temporarily in the liver and serum from 8 to 24 h after intravenous injection of Con A (13 mg/kg), and they could be detected again at 48 h after recovery from hepatic injury.^8^ Likewise, in hepatectomy‐induced hepatic injury model, the expression of either LECT2 mRNA or LECT2 protein could not be detected in the liver tissue at 0.5 h after partial hepatectomy. A significant magnitude of hepatocytes expressing LECT2 mRNA and protein could be seen across the liver at 6 h.^9^ It was observed in patients with acute liver failure that the level of serum LECT2 was lower in the expired group, compared with the alive group (0.96 ± 0.8 ng/ml vs 12.9 ± 4.3 ng/ml *p* < 0.05). Serum LECT2 level increased with the improvement of liver function. Besides, it was negatively correlated with serum AST(aspartate transaminase) and ALT (alanine transaminase) level, the nadir of LECT2 was concomitant with the peak of ALT.^10^

All these studies suggest that LECT2 may participate in hepatic regeneration in the early phase.

### 1.2 Neuronal development

Koshimizu et al^11^ uncovered that the length of axons and dendrites was shorter in neurons from LECT2‐knockout mice after 4 days of culture in vitro. Moreover, the neurons from LECT2‐knockout mice had several neurotrophin expressions that differed from that of wild‐type mice, including NGF (nerve growth factor), BDNF (brain‐derived neurotrophic factor) and NT‐3 (neurotrophin‐3). A subsequent investigation^12^ elucidated that there were more fragmentations and shorter microtubules in cultured neurons from LECT2‐knockout mice. Furthermore, an increased expression of katanin‐P60, a microtubule‐severing enzyme, was present in these cultured neurons at the first and the fourth day, when compared to the wild‐type ones.

The results show that LECT2 may affect the extension and neurotrophin expressions of the brain neurons during neuronal development. Yet, the function of LECT2 in the brain remains to be deeply explored and more studies are expected to spring up.

### 1.3 HSC homeostasis

Lu et al^13^ illustrated that TNF(tumour necrosis factor) was the specific downstream target of LECT2 signal in the macrophages and osteolineage cells. Recombinant LECT2 administration were capable of enhancing HSC (haematopoietic stem cells) expansion in the marrow and promoting HSC mobilization to the blood, on account of reduced TNF expressions from these macrophages and osteolineage cells through LECT2/CD209a axis. The effect of LECT2 on HSC fell into a decline, after a specific depletion of macrophages and osteolineage cells, or in the TNF‐knockout mice.

The study may bring a promising future for the treatment of patients who are in need of HSC transplantation due to a wide variety of haematological diseases.

### 1.4 Liver injury

LECT2 may play a role in the pathogenesis of liver injury. Saito et al^14^ found that the proportion of hepatic NKT cells was increased in the LECT2‐deficient mice (C57BL/6J genetic background) and the ConA‐induced hepatic injury was exacerbated in the LECT2‐deficient mice when treated with a NKT cell activator termed α‐galactosylceramide, owing to the over‐expressions of IL‐4 and Fas ligand.

Subsequently, Okumura et al^15^ reported that the amount of NKT cells within the liver varied with the genetic background of mouse strain. It made no significant difference between the LECT2‐deficient mice (BALB/c genetic background) and the wild‐type mice in the number of hepatic NK and NKT cells. Lipopolysaccharide/D‐galactosamine (LPS/D‐GalN)‐induced acute liver injury was alleviated in the LECT2‐deficient mice, as a result of significantly decreased IFN‐γ (interferon‐γ) production from NK and NKT cells in the liver.

These findings indicate that LECT2 could affect the functions of immune response cells and it may be involved in the pathogenesis of hepatitis.

### 1.5 Liver fibrosis

Xu et al^16^ discovered that a higher level of serum LECT2 was present in patients with liver fibrosis, which was closely correlated with liver fibrosis staging. LECT2 was identified as a functional ligand of Tie1 on endothelial cells, the direct interaction between LECT2 and Tie1 was able to suppress migration ability and tube formations of the endothelial cells in vitro. LECT2 over‐expression inhibited portal angiogenesis, promoted liver sinusoid capillarization and worsened the liver fibrosis in vivo. Nevertheless, these variations could be reversed in LECT2‐knockout mice. Subsequent study^17^ suggested that AAV9‐LECT2 small hairpin RNA in combination with either bevacizumab or rVEGF could improve therapeutic effects on liver fibrosis. Yet, AAV9‐LECT2 small hairpin RNA combining with bevacizumab showed better therapeutic outcomes and less side effects than with rVEGF in mice.

The expressions of serum LECT2 may vary in different pathogenesis, with the advancement of liver fibrosis. Xu et al^18^ found that there was a positive relationship between LECT2 and the progression of liver fibrosis. Additionally, when acting as a direct biomarker to predict liver fibrosis in patients (*n* = 147) with CHB (chronic hepatitis B), LECT2 was superior to the most common noninvasive scoring systems such as APRI (aspartate aminotransferaseto‐platelet ratio index) and FIB‐4 (fibrosis index based on the four factors). However, Sak et al^19^ observed that the level of LECT2 in patients with alcohol‐induced liver cirrhosis decreased with the progression of cirrhosis. The concentrations of serum LECT2 decreased to 11.06 ± 6.47 ng/ml in the Pugh‐Child A + B group (*n* = 37 *p* < 0.0001), then dropped to 8.06 ± 5.74 ng/ml in the Pugh‐Child C group (*n* = 32 *p* < 0.0001), compared with the control group (18.99 ± 5.36 ng/ml n = 17).

In brief, the referred researches illustrate that LECT2 may play a crucial role in liver fibrogenesis, and it may represent a potential biomarker and therapeutic target for liver fibrosis.

### 1.6 Hepatocellular carcinoma

Generally, LECT2 expressions were strikingly down‐regulated in human HCC (hepatocellular carcinoma) samples (78.4%, 40/51), relative to normal liver tissues.^20^ Weaker LECT2 expression synchronized with higher histological grade of HCC and further progression of multistep hepatocarcinogenesis.^21^ In Ctnnb‐1 mutated HCC model, the absence of LECT2 in tumour hepatocytes contributed to the EMT (epithelial to mesenchymal transition) of cancer cells and the recruitment of immature inflammatory monocytes that possessed immunosuppressive properties and tumour‐promoting potential.^22^

Despite a lower LECT2 expression in HCC samples, a relatively higher level of serum LECT2 was observed in HCC patients than in patients with cirrhosis or healthy volunteers. Serum LECT2 level could serve as a diagnostic biomarker for HCC, with a sensitivity of 59.3%, specificity of 96.1%, positive predictive value of 97.0% and negative predictive value of 53.2%, respectively, based on the cut‐off value at 50 ng/ml.^23^

In addition, LECT2 could directly bind to VEGFR2(vascular endothelial growth factor receptor2) to suppress tumour growth via decreased angiogenesis and down‐regulate VEGF_165_‐induced VEGFR2 tyrosine phosphorylation and downstream protein signalling.^24^ Moreover, LECT2 could also directly interacted with the MET receptor at the cell membrane, antagonizing its activation through the recruitment of protein tyrosine phosphatase 1B, leading to the MET dephosphorylation. Clinical evidence indicated that longer survival time and less vascular invasions could be observed in patients with higher LECT2 expressions and lower phosphorylated MET levels in HCC samples.^25^, ^26^

The mentioned results reveal that LECT2 may take part in hepatocarcinogenesis and this may open a potential avenue, in terms of diagnosis and treatment, for HCC.

### 1.7 Metabolic disorders

Serum LECT2 levels were positively correlated with liver triglyceride contents in mice,^27^ and plasma LECT2 levels exhibited a strong relationship with visceral fat area in human.^28^ Higher levels of LECT2 expression were present in participants with dyslipidemia and plasma LECT2 levels could be used as potential biomarker to differentiate the participants with dyslipidemia from those without dyslipidemia, accompanied by a sensitivity of 60.3%, specificity of 66.7%, based on the plasma cut‐off value at 16.5 ng/ml.^28^ In parallel with the above studies, the increase in serum LECT2 levels was also found in population with obesity and fatty liver, which was positively correlated with all the four major anthropometric measures in both males and females, including BMI (body mass index), WC (waist circumference), WHR (waist‐to‐hip ratio) and W/Ht (waist‐to‐height ratio).^29^

Serum LECT2 levels presented a close correlation with the severity of both obesity and insulin resistance in human. It was validated in mice that HFD (high‐fat diet) led to increased LECT2 expressions that were derived from decreased AMPK phosphorylations in hepatocytes and the overproduced LECT2 attenuated insulin signal transduction via increased JNK phosphorylations in skeletal muscle cells. Improved muscle insulin sensitivity could be observed in LECT2‐deficient mice.^30^ Gemigliptin, a dipeptidyl peptidase‐4 (DPP‐4) inhibitor, was able to relieve hepatic steatosis and insulin resistance by suppressing LECT2 expressions involved in increased AMPK phosphorylations in HFD‐fed mice.^31^ A recent study demonstrated that LECT2 mRNA levels were positively associated with the mRNA levels of the inflammation‐related genes CCR2 and TLR4. Moreover, LECT2 contributed to M1‐like macrophage phenotype and the M1/M2 ratio, making a linkage from liver steatosis to hepatic inflammation in NASH.^32^

LECT2 may mediate the pathogenesis and progression of atherosclerosis. In HUVECs and THP‐1 cells, LECT2 administrations were sufficient to promote the expressions of ICAM‐1 (intercellular adhesion molecule‐1) and several pro‐inflammatory cytokines, including TNFα, MCP‐1 (monocyte chemo‐attractant protein‐1) and IL‐1β (interleukin‐1β), relying on CD209/JNK signalling.^33^ Besides, in 3T3‐L1 adipocytes, incremental adipose synthesis, impaired insulin signalling and induced inflammatory response had been achieved via CD209/P38 and JNK‐mediated pathway, when treated with LECT2.^34^

Collectively, as a novel obesity‐related protein, LECT2 might directly participate in the inflammatory reactions in human endothelial cells. Meanwhile, it may be considered as a potential biomarker and therapeutic approach for obesity and diabetes.

### 1.8 Inflammatory arthritides

RA(rheumatoid arthritis) is a chronic, inflammatory and autoimmune disease that can cause a progressive destruction of joint cartilage. The polymorphism of LECT2 genotype was clearly associated with the incidence and severity of RA in the Japanese population. Individuals with 1 Ile58 allele were more likely to develop RA, while those with 2 Ile58 alleles were more prone to severe disease.^35^ LECT2 was found identical to chondromodulin II which was extracted from fetal bovine cartilage, and in vitro, it was potent to stimulate the proteoglycan synthesis of rabbit growth plate chondrocytes and promote the proliferation of primary mouse osteoblasts.^36^, ^37^ It has been verified in anti‐type II collagen antibody‐induced arthritis model that LECT2‐deficient mice suffered from aggravated arthritis, which was evidenced by serious swollen hind paws, incremental cartilage erosion and increased inflammatory cytokines such as IL‐1β and IL‐6, compared with the wild‐type controls. The severity of arthritis could be ameliorated by exogenous LECT2 protein.^38^

Together, these data uncover that LECT2 may be utilized as a novel therapeutic option regarding inflammatory arthritides such as RA.

### 1.9 Systemic sepsis

LECT2 enhanced *Helicobacter pylori* elimination in RAW264.7 macrophages and intensified the phagocytic effect and bacterial killing of macrophages in septic mice via CD209a receptor.^39^, ^40^ Administration of exogenous LECT2 enabled the wild‐type septic mice to survive longer via down‐regulation of TNF‐α and IL‐6, compared with the LECT2‐deficient septic mice.^41^ A clinical study^42^ indicated that the LECT2 concentrations negatively correlated with the CRP (C reactive protein) concentrations as well as the severity of systemic inflammation, in patients with sepsis. The LECT2 concentrations were significantly lower in the septic patients at ICU entry (5.3 ± 4.1 ng/ml), in contrast with themselves at ICU discharge (13.2 ± 4.9 ng/ml), but the levels of LECT2 were still lower than those of the healthy volunteers (19.7 ± 3.4 ng/ml).

These studies highlight the potential value of LECT2 as a diagnostic indicator and immunal modulation target for acute severe inflammation.

### 1.10 Systemic amyloidosis

Amyloidosis is the abnormal deposition and aggregation of insoluble protein fibrils in tissues, leading to progressive organ dysfunction. In addition to the liver,^43^ the kidney is the site most frequently affected by systemic amyloidosis. Currently, more than 30 proteins have been found to cause amyloidosis in human.^44^ First described in 2008, LECT2 is a newly discovered amyloidogenic protein in a renal biopsy.^45^

LECT2 amyloidosis (ALECT2) accounted for 2.5% (7/285) and 2.7% (13/474) cases in two large series of renal amyloidosis, respectively.^46^, ^47^ ALECT2 has a strong racial bias and it is highly prevalent in the Hispanic population. Among the patients with ALECT2, Hispanics made up a considerable percentage of 88% (35/40) and 92% (66/72) cases respectively in two studies focusing on renal amyloidosis.^48^, ^49^ Genomic analysis indicated that no pathogenic mutations were detected in the LECT2‐encoding gene, although each of the patient was homozygous for the G allele (SNP rs31517 in exon 3), which was present in about 60% of the European population.^43^, ^48^, ^50^, ^51^

An observational study (NCT03774784) has been launched to investigate the natural history of ALECT2. As yet, there is no better therapy except transplantation for ALECT2. Recently, it has been suggested that I40V mutation may destabilize the structure of LECT2 and increase the aggregation tendency, when in a deficiency or removal of zinc icon.^52^ Whereas, LECT2 amyloidosis remains unclear and elusive, more thorough studies are in need to explore the disease.

## 2 DISCUSSION AND PERSPECTIVES

LECT2 is mainly produced and released to blood by hepatocytes, like albumin, it is able to reflect the condition of liver function at certain degree. From the prognostic point of view, serum LECT2 level may represent a good indicator for some acute critical liver‐related diseases, including acute fulminant hepatitis and acute liver failure. Not confined to acute critical liver‐related disease, LECT2 is of great value in the detection of chronic hepatic disease. Higher expressions of serum LECT2 were detected in patients with liver fibrosis, and they were closely correlated with liver fibrosis staging, even if these patients were within normal serum ALT levels. LECT2, as a latest ligand for Tie1, played a pro‐fibrogenic role in the progression of liver fibrogenesis. LECT2‐Tie1 signal promoted liver sinusoid capillarization and inhibited portal angiogenesis, thereby worsening the liver fibrosis. Whereas, these variations could be reversed in LECT2‐knockout mice. Previous concrete studies indicated that sinusoidal angiogenesis was necessary for the resolution of fibrosis.^53^, ^54^, ^55^ Thus, LECT2 may be used as a noninvasive diagnostic marker and potential therapeutic target for liver fibrosis.

Chronic fibrosis drives the pathological condition to develop into liver cirrhosis, resulting in a rising incidence of HCC. HCC is a type of hypervascular tumour featured by tremendous angiogenesis, and the neovascularization contributes to the growth and metastasis of the tumour, giving rise to a dismal prognosis.^56^, ^57^ LECT2 could not only inhibit VEGF_165_‐induced angiogenic effects via VEGFR2, but also exert a specific suppressive effect on HCC growth and vascular invasion through the Met receptor. Longer survival time and less vascular invasions were present in HCC patients with higher LECT2 expressions and lower phosphorylated MET levels in HCC specimens. In these regards, LECT2 may serve as a candidate prognostic biomarker and therapeutic target for HCC as well.

LECT2 exhibited a close correlation with obesity and obesity‐related liver diseases in both males and females, and it could draw a clear distinction between the subjects with or without fatty liver. In mice, HFD enabled the increase in the levels of LECT2, and the overproduced LECT2 further impaired insulin sensitivity and induced insulin resistance in skeletal muscle cells. Fortunately, Gemigliptin, a novel DPP‐4 inhibitor, could efficiently inhibit LECT2 expressions, improve hepatic steatosis and ameliorate insulin resistance by increasing AMPK phosphorylation and reducing CD209 receptor‐mediated JNK pathway. This means LECT2 plays an important role in the regulation of insulin sensitivity, and it may provide a novel perspective for the therapy of obesity‐associated insulin resistance.

The balance between pro‐inflammatory and anti‐inflammatory cytokines has a direct impact on the severity of inflammation. IFN‐γ, as a pro‐inflammatory cytokine, is primarily produced by activated lymphocytes, including CD4 and CD8 T cells, gamma delta T cells and NK and NKT cells.^58^ The production of IFN‐γ from these cells could also stimulate macrophages to produce several pro‐inflammatory cytokines, such as TNF‐α.^59^, ^60^ In LECT2‐deficient mice, LPS/D‐GalN‐induced acute liver injury was alleviated, resulting from decreased IFN‐γ levels. LECT2 may take direct participation in the functional regulation of NK and NKT cells, affecting the production of IFN‐γ. Therefore, it may be reasonable to assume that LECT2 involves in the pathogenesis of human inflammatory disorders, and it may be a useful target for these diseases, such as autoimmune hepatitis and rheumatoid arthritis.

Sepsis, characterized by a hyper‐inflammatory response due to a systemic microbial infection, is one of the most common causes of death among critically ill patients in the intensive care unit.^61^ CRP concentrations are often used as an indicator to reflect the severity of sepsis for the reason that increased CRP levels exhibit a close association with high infections and mortality rates in critically ill patients.^62^, ^63^ LECT2 exerts a repressive effect on the excessive inflammation and it shows a strong and negative correlation with CRP. In this regard, LECT2, analogous with CRP, may act as a reliable diagnostic biomarker indicative of the severe inflammation. But LECT2 may not suitable to predict mortality among the patients with sepsis and organ failure, since no significant correlation was present between LECT2 and APACHEII (acute physiology and chronic health evaluationII) and SOFA (sequential organ failure) scores.

G‐CSF(granulocyte colony stimulating factor), as a bone marrow (BW)‐derived cytokine, is a HSC‐mobilizing agent widely used in clinical settings. For some cancer patients undergoing chemotherapy or radiotherapy, it may fail to mobilize HSC due to a severe alteration of the BM microenvironment induced by G‐CSF.^64^, ^65^ LECT2, as an extra‐medullar cytokine, remained to take effects on HSC‐mobilizing and expansion in the irradiated mice, while G‐CSF did not. LECT2 differed from G‐CSF in the mechanism of inducing HSC expansion and mobilization. It caused an increase in the number of macrophages, without changing the amount of osteolineage cells, whereas G‐CSF brought about a decrease in both macrophages and osteolineage cells in the BM. There was no synergistic effect when it was used in combination with G‐CSF. Thus, LECT2 may represent a potential HSC‐mobilizing agent in those patients who do not exhibit mobilization with G‐CSF treatment.

**FIGURE 1:**
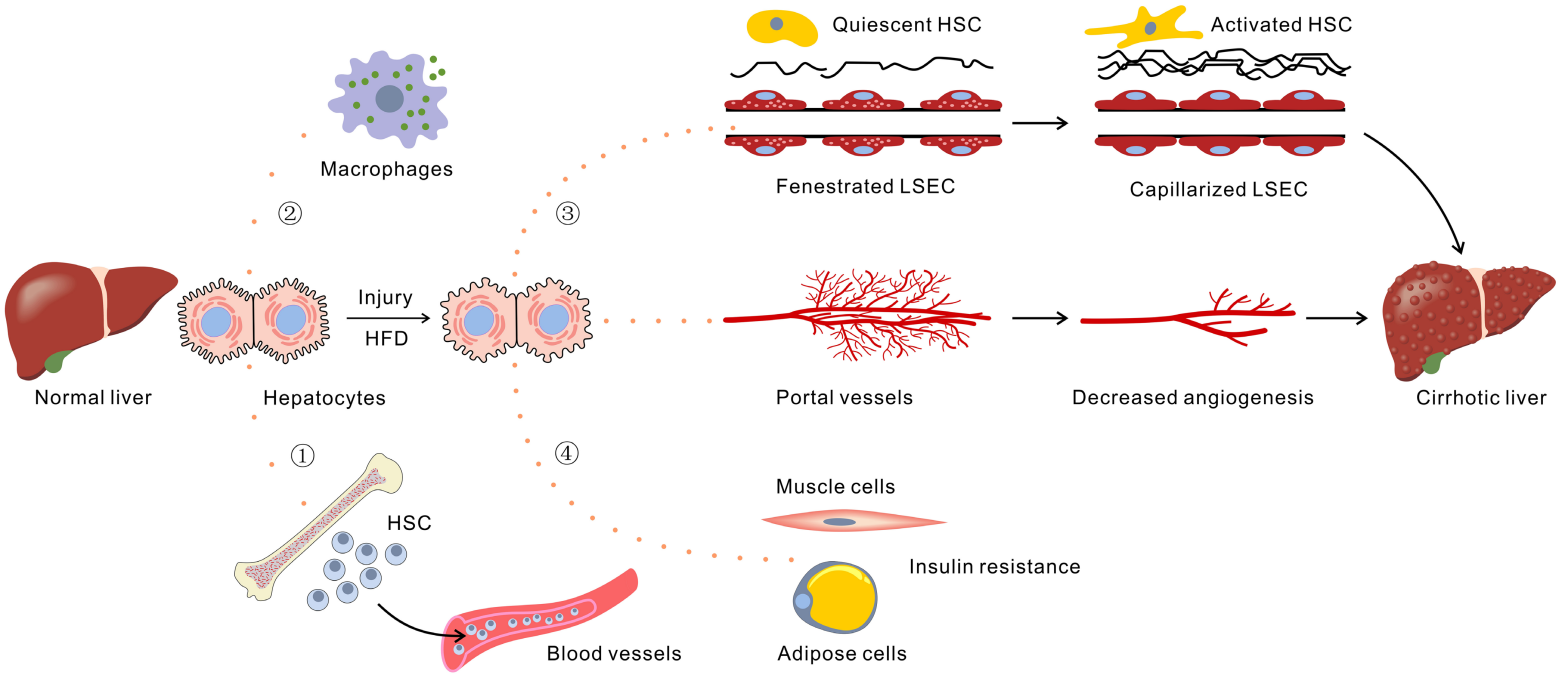
The effect of LECT2 on various cells in vivo. HSC (haematopoietic stem cells; hepatic stellate cells) ① A proper level of LECT2 stimulus enables the haematopoietic stem cells to expand in the bone marrow and mobilize to the blood. ② An appropriate level of LECT2 stimulus strengthens the phagocytic effect and bacterial killing of macrophages. ③ Chronic hepatic injury enhances the expression of LECT2 and the overexpressed LECT2 promotes the capillarization of LSEC (liver sinusoidal endothelial cells) and suppresses the angiogenesis of portal area, worsening liver fibrosis as a result. ④ HFD (high‐fat diet) increases the expression of LECT2 and the excessive LECT2 impairs the insulin sensitivity of the skeletal muscle and adipose cells, contributing to insulin resistance as a consequence

In general, LECT2 is a pleiotropic and promising hepatokine, it seems to function a lot in almost every system in the human, and it plays diverse and pivotal roles in different physiological conditions and pathological abnormalities (Figure 1). LECT2 may serve as a novel molecular biomarker and therapeutic target for these diseases in the near future, with great value of clinical application. Certainly, more basic studies are needed to clarify the exact relationship and mechanism between LECT2 and these diseases, meanwhile more clinical trials are required to validate the efficacy of clinical application.

## AUTHOR CONTRIBUTIONS

**Yuan Xie:** Writing – original draft (lead). **Kai‐Wei Fan:** Writing – original draft (equal). **Shi‐Xing Guan:** Data curation (supporting). **Yang Hu:** Data curation (supporting). **Yi Gao:** Writing – review and editing (equal). **Wei‐Jie Zhou:** Writing – review and editing (lead).

## CONFLICT OF INTEREST

We declare no conflict of interest in this work, and all authors approve the manuscript.

## Data Availability

Data sharing is not applicable as no new data were generated for the review article.
